# A newly identified Hippo homologue from the oriental river prawn *Macrobrachium nipponense* is involved in the antimicrobial immune response

**DOI:** 10.1186/s13567-021-00945-7

**Published:** 2021-06-02

**Authors:** Ying Huang, Qian Ren

**Affiliations:** 1 College of Oceanography, Hohai University, 1 Xikang Road, Nanjing, 210098 Jiangsu China; 2 College of Marine Science and Engineering, Nanjing Normal University, 1 Wenyuan Road, Nanjing, 210023 Jiangsu China

**Keywords:** *Macrobrachium nipponense*, Hippo, Expression regulation, Antibacterial response, Innate immunity

## Abstract

The Hippo signalling pathway plays a vital role in organ size control, cell proliferation, apoptosis, and immune regulation. In this study, a Hippo homologue with three isoforms (named *MnHippo-a*, *MnHippo-b*, and *MnHippo-c*) was isolated and characterized for the first time from the freshwater prawn *Macrobrachium nipponense*. The deduced amino acid sequences of MnHippo-a (698 aa), MnHippo-b (688 aa), and MnHippo-c (656 aa) were highly similar, and they all contained an N-terminal S_TKc (serine/threonine protein kinase catalytic) domain and a C-terminal Mst1_SARAH (Sav/Rassf/Hpo) domain. *MnHippo-a* and *MnHippo-c* were derived from alternative splicing. Phylogenetic analysis was performed, and the results revealed that MnHippo was a member of the clade containing STPK4 and Hippo of *Penaeus vannamei*. The expression distribution showed that *MnHippo* was constitutively expressed in various tissues of uninfected prawns and highly expressed in the hepatopancreas and intestine. In prawns challenged with *Vibrio parahaemolyticus* and *Staphylococcus aureus*, the expression of *MnHippo* in haemocytes was significantly upregulated. Furthermore, in *MnHippo*-knockdown prawns injected with *V. parahaemolyticus* or *S. aureus*, the transcription levels of five antimicrobial peptides were downregulated. *MnHippo* silencing weakened the clearance of *V. parahaemolyticus* and *S. aureus* in prawns. The survival rate of the *MnHippo*-dsRNA group was obviously decreased from 2 to 6 days post-injection with *V. parahaemolyticus* or *S. aureus*. Hence, *MnHippo* might be involved in the antibacterial immune defence of *M. nipponense*.

**Supplementary Information:**

The online version contains supplementary material available at 10.1186/s13567-021-00945-7.

## Introduction

The Hippo signalling pathway is found in a wide variety of animals, from insects to mammals. Since its initial discovery in *Drosophila melanogaster*, the Hippo pathway has attracted much attention because of its strong involvement in controlling organ size, regulating cell proliferation, and promoting apoptosis [1–3]. The Hippo pathway is composed of more than 30 components, including the core kinase module and the transcriptional module [4]. The core members of this pathway were identified through genetic screening of *Drosophila* chimaeras and include four proteins, namely, Hippo (Hpo), Salvador (Sav), Warts (Wts), and monopolar spindle one binder (MOB), as tumour suppressors (Mats) [5]. Under activator stimulation (the Hippo pathway is activated), the four proteins can form a kinase cascade reaction chain. Hpo is phosphorylated first, and activated Hpo phosphorylates a complex composed of Wts and the adaptor protein Mats (Wts-Mats) with the help of Sav. The Hippo pathway kinase cascade in turn promotes the inhibitory phosphorylation of the transcriptional co-activator Yorkie (Yki) and excludes Yki from the nucleus to promote apoptosis and restrict the overgrowth of organ size [6–8]. When the Hippo pathway is inactivated, unphosphorylated Yki translocates into the nucleus, where it binds to and thus activates the transcriptional partner Scalloped (Sd), which contains a TEA domain [9]. The Yki-Sd complex promotes tissue overgrowth by acting on downstream target genes, such as connective tissue growth factor (CTGF) and cysteine-rich 61 (Cyr61) [10]. The loss of functional mutations in any of the four genes (Hpo, Sav, Wts, and Mats) or overexpression of Yki will lead to excess growth because of increased cell proliferation coupled to decreased cell death [11].

Although the Hippo pathway is an extremely complex signalling network, it is highly conserved throughout the evolutionary process. Most members of the *Drosophila* Hippo pathway have homologues in mammals. Hpo, Sav, Wts, Mats, and Yki in *Drosophila* are homologous proteins of mammalian Sterile20-like protein kinase 1/2 (Mstl/2, also known as STK4/3), Salvador family WW domain-containing protein 1 (Savl), large tumour suppressor kinase 1/2 (Lastl/2), MOB kinase activator 1A/B (MoblA/B), and yes-associated protein (YAP)/transcriptional co-activator with PDZ-binding motif (TAZ, also known as WWTR1), which are all functionally conserved [10, 12]. Recent research using multiple model systems showed that the Hippo kinase cascade represents a signal transduction module that integrates multiple biological inputs, thereby highlighting its importance in biological growth control [13]. Yki in *Drosophila* or YAP/TAZ in mammals is the target molecule of the Hippo pathway, and regulation of this pathway is ultimately attributed to the regulation of Yki or YAP/TAZ [7, 14]. Phosphorylation of the YAP/TAZ protein controls the size of the mouse liver, while overexpression of YAP/TAZ leads to overgrowth of the liver and induces tumorigenesis [12, 15]. In addition to its roles in tissue development and tumorigenesis, the Hippo pathway also has extensive functions in immune regulation, including of the innate and adaptive immune systems [16]. For example, *Drosophila* Hpo and its mammalian homologue Mstl/2 mediate Toll-like receptor (TLR) signalling in flies [17] and mammals [18, 19]. YAP/TAZ binds and inhibits TANK binding kinase 1 (TBK1) or interferon regulatory factor 3 (IRF3) to antagonize antiviral innate immune responses [20, 21]. Although the function of the Hippo pathway has been extensively studied in *Drosophila* and most mammals, the roles of the homologous members of the pathway in the immune system of crustaceans remain unclear.

*Macrobrachium nipponense* is an economically important freshwater prawn widely cultivated in China, Japan, and Southeast Asian countries [22]. Whether the Hippo signalling pathway plays a role in the innate immunity of *M. nipponense* remains unknown. In the present study, we isolated a Hippo homologue with three isoforms from *M. nipponense* (named *MnHippo-a*, *MnHippo-b*, and *MnHippo-c*) and analysed their sequence features. The tissue distribution profile and temporal mRNA expression of *MnHippo* in response to bacterial infection were detected. RNA interference (RNAi) was implemented to determine the function of *MnHippo* in antibacterial responses. The results of our work will provide insights into the immune defence mechanism of the Hippo pathway in crustaceans.

## Materials and methods

### Experimental animals

Healthy *M. nipponense* prawns (approximately 2.5–3.5 g in weight) were obtained from a commercial market in Nanjing city, China. The prawns were cultured at 24 °C–26 °C in a recirculating aquarium system filled with freshwater. After 10 days of acclimatization in our laboratory, the haemocytes, heart, hepatopancreas, gills, intestine, and stomach were dissected from five prawns. The samples were quickly frozen in liquid nitrogen and stored at −80 °C until RNA extraction.

### Bacterial challenge

Ninety prawns were divided into three groups, namely, the *Vibrio parahaemolyticus* group, *Staphylococcus aureus* group, and control group. In the two challenged groups, each prawn was injected with 50 μL of *V. parahaemolyticus* (3 × 10^7^ cells) and *S. aureus* (3 × 10^7^ cells). Prawns in the control group were injected with 50 μL of phosphate-buffered saline (PBS; 140 mM NaCl, 2.7 mM KCl, 10 mM Na_2_HPO_4_, 2 mM KH_2_PO_4_; pH 7.4). The prawns were returned to the rearing tanks. At 0, 12, 24, 36, and 48 h post-injection, haemolymph was randomly collected from the ventral sinus of five prawns in the control and experimental groups. The haemolymph was extracted using a 1 mL syringe, mixed with a 1/2 volume of anticoagulant solution (ACD-B) (glucose, 1.47 g; citric acid, 0.48 g; trisodium citrate, 1.32 g; prepared in ddH_2_O added up to 100 mL; pH 7.3), and centrifuged at 2000 *g* and 4 °C for 10 min to harvest haemocytes. The haemocyte pellets were stored at −80 °C for subsequent experiments.

### RNA extraction and reverse transcription

Total RNA was isolated from the above tissue samples by using TRIzol Reagent (Invitrogen, USA). The quality and concentration of total RNA were examined by RNA electrophoresis and a NanoDrop 2000 Spectrophotometer (Thermo Scientific, USA). All RNA samples were added to RNase-free Dnase I (Takara, Japan) to eliminate genomic DNA. In brief, 5 µg of RNA from the mixed tissues was reverse transcribed to first-strand cDNA by using the SMARTer™ RACE cDNA Amplification Kit (Clontech, USA). Total RNA (1 μg) was reverse transcribed using the PrimeScript® 1st Strand cDNA Synthesis Kit (Takara, Japan) for real-time PCR analysis. The obtained cDNA was kept at −20 °C.

### Molecular cloning of *MnHippo*

The partial fragment of *Hippo* cDNA was obtained from the transcriptomic cDNA library of gills of *M. nipponense* from our laboratory. Two gene-specific primers (*MnHippo*-F and *MnHippo*-R, Table 1) were designed based on the obtained cDNA sequence to carry out rapid amplification of the cDNA ends (5ʹ and 3ʹ RACE). 5ʹ and 3ʹ RACE was performed using an Advantage® 2 PCR Kit (Clontech, USA) according to the protocol. PCR amplification was performed in a 50 μL reaction as follows: five cycles at 94 °C for 30 s and 72 °C for 3 min; 5 cycles of 94 °C for 30 s, 70 °C for 30 s, and 72 °C for 3 min; 20 cycles of 94 °C for 30 s, 68 °C for 30 s, and 72 °C for 3 min. The resulting PCR products were electrophoresed on a 1.2% agarose gel and purified with a gel extraction kit (Takara, Japan). The expected purified fragments were sub-cloned into the pEasy-T3 vector (TransGen Biotech, China), and positive clones were selected and sequenced (Springen, China). The 5ʹ and 3ʹ sequences from RACE were assembled with the partial cDNA sequence to obtain the complete sequence of *MnHippo,* as shown in Additional file 1.

**Table 1:** **Primer sequences used in this study**

Primer name	Sequence (5ʹ-3ʹ)
*MnHippo*-F	GATTCGTTCTCCCAGCAGCAGTCCCCTC
*MnHippo*-R	TCTGGTTGCCACTGGTCAGGCTCACG
*MnHippo-*gF1	TGACACTGACCTTCAGGAAATC
*MnHippo-*gR1	AATACTCTAGGCCACGCAAAG
*MnHippo-*gF2	AGATAGCGACAGTTATCCTA
*MnHippo-*gR2	ACTCCATATATCTGCAACAC
*MnHippo-*gF3	GCTGGTAACATTCTGCTAAATACTC
*MnHippo-*gR3	GTGATGTGCTTGATTTGGATCTG
*MnHippo-*gF4	GAACTCCTCCAACACCAGTTTA
*MnHippo-*gR4	TGTTAGGTATGCTGTGGTGATG
*MnHippo-*gF5	GTACAGGTCTGGAGGAGATAGT
*MnHippo-*gR5	CCTGGAGCTGTATCATGTCTTT
*MnHippo*-qF	TTTGCGTGGCCTAGAGTATTT
*MnHippo*-qR	CGCATGGCCCTGAGTATTTA
*β*-actin-qF	TATGCACTTCCTCATGCCATC
*β*-actin-qR	AGGAGGCGGCAGTGGTCAT
*MnHippo*-RT-F	GGTGACTTCCAGGGTGAAATAA
*MnHippo*-RT-R	CCACGCAAAGTGTCTAGGATAA
*β*-actin-RT-F	AATGTGTGACGACGAAGTAG
*β*-actin-RT-R	GCCTCATCACCGACATAA
*MnHippo*-dsRNA-F	GCGTAATACGACTCACTATAGGGAGCAAGAAAAGAAGGC
*MnHippo*-dsRNA-R	GCGTAATACGACTCACTATAGGCTGCAGCAACTCCTCTTG
GFP-dsRNA-F	GCGTAATACGACTCACTATAGGTGGTCCCAATTCTCGTGGAAC
GFP-dsRNA-R	GCGTAATACGACTCACTATAGGCTTGAAGTTGACCTTGATGCC
*MnALF1*-qF	GTGGTGCCCAGGATGGACTT
*MnALF1*-qR	AGAGGATGGTGGAGGAAATT
*MnALF4*-qF	GGCAGAGGGCCAAGAATTAG
*MnALF4*-qR	GAATTCCAAGTCACCTGTCTCC
*MnCru5*-qF	ACACCCCAATCACCCCCCA
*MnCru5*-qR	TGCCTTGAAACGGCTCCCT
*MnCru7*-qF	GACCTGCCTGTCCCCCCGT
*MnCru7*-qR	CCCCACACCTCAGCCCAAA
*MnLyso2*-qF	GGCAAGACAGAAAGAGAGAGAG
*MnLyso2*-qR	GAGACCGCTGAAACCACTAAA

### Genome sequence amplification

Genomic DNA was extracted from the gills of healthy prawns by using NucleoSpin Tissue (Clontech, USA). Genome amplification was performed in a 25 μL reaction volume containing 18.3 μL of sterile distilled H_2_O, 2.5 μL of 10 × PCR buffer, 2.0 μL of dNTPs (2.5 mmol L^−1^), 0.5 μL of each primer (10 μmol L^−1^), and 0.2 μL of Taq polymerase (5 U μL^−1^) (Takara, Japan). PCR amplification was conducted as follows: 94 °C for 3 min; 30 cycles of 94 °C for 30 s, 53 °C for 45 s, and 72 °C for 3 min; and 72 °C for 10 min. The detailed methods were described in our previous study [23]. The primer sequences (MnHippo-gFs and MnHippo-gRs) are shown in Table 1.

### Nucleotide and amino acid sequence analysis

Searches for protein sequence similarities were performed using the BLAST algorithm on the NCBI website. Protein prediction was carried out using the ExPASy Translate tool and the open reading frame (ORF) finder. Signal sequence and motif prediction was conducted with the SMART program. The molecular weight (Mw) and theoretical isoelectric point (pI) were calculated with the ExPASy Compute pI/Mw tool. The deduced amino acid sequences of Hippo homologues from *M. nipponense* and other species were compared by multiple sequence alignment using the ClustalX v2.0 program and GENEDOC software. A bootstrap neighbour-joining (NJ) phylogenetic tree was built using MEGA 7.0 software [24].

### Quantitative real-time PCR (qRT-PCR)

qRT-PCR was carried out using the TransStart® Top Green qPCR SuperMix Kit (TransGen Biotech, China) with the LightCycler® 96 Real-Time PCR Detection System (Roche, USA). Amplifications were conducted with a 10 μL reaction volume containing 5 μL of 2× TransStart Top Green qPCR SuperMix, 1 μL of cDNA template (50 ng), 0.2 μL of forward and reverse primers (10 μM), and 3.6 μL of ddH_2_O. The PCR temperature profile was as described in a previous study [25]. One pair of primers (*MnHippo*-qF and *MnHippo*-qR, Table 1) was designed to analyse the expression pattern of *MnHippo*. The reference gene used to normalize the expression of *MnHippo* was the *β*-actin gene of *M. nipponense* (*β*-actin-qF and *β*-actin-qR, Table 1). Each sample was analysed in triplicate. Differences in gene expression were calculated by the 2^−ΔΔCt^ method [26]. The data are presented as the mean ± S.D. (*N* = 5). An unpaired sample *t*-test was used for statistical analysis, and differences were considered significant if *P* < 0.05.

### Semiquantitative reverse transcription-PCR (SqRT-PCR)

*MnHippo* expression in different tissues was investigated by SqRT-PCR using two primers (*MnHippo*-RT-F and *MnHippo*-RT-R, Table 1). The PCR amplification procedure was as follows: 94 °C for 3 min; 30 cycles of 94 °C for 30 s, 53 °C for 45 s, and 72 °C for 30 s; and 72 °C for 5 min. *β*-Actin was amplified as an internal control (*β*-actin-RT-F and *β*-actin-RT-R, Table 1). The PCR products were separated on 1.2% agarose gels with 0.5 µg/mL ethidium bromide and photographed under ultraviolet light using Quantity One software (Bio-Rad, Hercules, CA, USA).

### Double-stranded RNA (dsRNA) synthesis

DsRNA of *MnHippo* was synthesized using a T7 in vitro transcription kit (Takara, Japan) according to the manufacturer’s instructions. The green fluorescent protein (GFP) gene was used as a control. Primers used in RNA interference (RNAi) were designed according to the cDNA sequences of *MnHippo* and GFP. Templates were prepared by PCR using gene-specific primers (*MnHippo*-dsRNA-F and *MnHippo*-dsRNA-R; GFP-dsRNA-F and GFP-dsRNA-R, Table 1) with the T7 polymerase promoter sequence at their 5’ ends. The mixture consisted of 10 μL of 5× transcription buffer, 2.5 μL of each NTP (100 mM), 80 U of RNase inhibitor, 8 μg of DNA template, and 80 U of T7 RNA polymerase and was incubated at 37 °C for 6 h. The mixture was then treated with RNase-free DNase I (8 U) to eliminate the DNA template. The synthesized dsRNA was purified by phenol/chloroform extraction and ethanol precipitation. The product was dried and dissolved in RNase-free water. The quality of dsRNAs was assessed by 1.5% agarose gel electrophoresis and spectrophotometry. The dsRNA sample was kept at −80 °C until use.

### DsRNA interference of *MnHippo* and detection of antimicrobial peptide (AMP) expression

Twenty healthy prawns were assigned to two groups, namely, the *MnHippo*-dsRNA and GFP-dsRNA (as control) injection groups, to verify the efficiency of RNAi. Each prawn was initially injected with 15 μg of *MnHippo*-dsRNA or GFP-dsRNA. After 12 h, 15 μg of *MnHippo*-dsRNA or GFP-dsRNA was repeatedly injected into the same prawn. At 36 h after the first dsRNA injection, the haemocytes of five prawns from each group were randomly collected. qRT-PCR was used to validate the knockdown of *MnHippo*.

At 36 h after two injections of *MnHippo*-dsRNA or GFP-DSRNA (total 30 μg), the prawns were challenged with *V. parahaemolyticus* (3 × 10^7^ cells) or *S. aureus* (3 × 10^7^ cells). At 24 h post-bacterial challenge, the haemocytes of five prawns from each group were sampled. The expression of *MnHippo* and five *AMP* genes, including *anti-lipopolysaccharide factor 1* (*ALF1*), *ALF4*, *crustin 5* (*Crus5*), *Crus7*, and *lysozyme* (*Lyso2*), was analysed in *MnHippo*-dsRNA-silenced prawns. The primers for the *AMPs* were as follows: *MnALF1*-qF and *MnALF1*-qR; *MnALF4*-qF and *MnALF4*-qR; *MnCru5*-qF and *MnCru5*-qR; *MnCru7*-qF and *MnCru7*-qR; *MnLyso2*-qF and *MnLyso2*-qR. All primers were listed in Table 1. Each experiment was repeated three times. Statistical analysis was performed using SPSS software. Statistical significance was determined by one-way ANOVA (*P* < 0.05).

### Bacteria clearance assay

We further investigated the role of *MnHippo* in *V. parahaemolyticus* or *S. aureus* clearance in vivo. *MnHippo*-dsRNA as a treatment or GFP-dsRNA as a control was injected into the prawns twice (total 30 μg) as mentioned above. At 36 h after the first dsRNA injection, 50 μL of *V. parahaemolyticus* (3 × 10^7^ cells) or *S. aureus* (3 × 10^7^ cells) was injected into the prawns. Haemolymph from five prawns for each sample was collected and mixed with ACD-B buffer at 15 min post-bacterial injection. After serial dilution with PBS, 50 μL of the haemolymph was loaded on Luria–Bertani agar plates and incubated at 37 °C overnight. The number of bacterial clones in the plate was counted. The experiment was repeated three times.

### *M. nipponense* survival rate test

The prawns were randomly divided into four groups (20 prawns per group) to examine whether *MnHippo* is involved in the anti-bacterial immune defence in vivo. The prawns were injected with 30 μg of *MnHippo*-dsRNA or GFP-dsRNA twice and then injected with 50 μL of *V. parahaemolyticus* (3 × 10^7^ cells) or *S. aureus* (3 × 10^7^ cells) at 36 h after the first injection. In a fixed time period of 1–6 days after the last challenge, the number of dead prawns in each group was monitored, and the cumulative survival rates of prawns were calculated. The experiments were repeated three times under the same conditions.

## Results

### Cloning and sequence analysis of *MnHippo*

Three contigs of *MnHippo* were identified from the transcriptome data of *M. nipponense*. These contigs are referred to as *MnHippo-a* (8022 bp), *MnHippo-b* (7992 bp), and *MnHippo-c* (7896 bp) on the basis of their nucleotide sequences (Additional file 1). The full-length cDNA sequence of *MnHippo-a* consisted of an ORF of 2097 bp encoding a protein of 698 amino acid residues, 136 bp of 5ʹ-untranslated region (UTR), and 5789 bp of 3ʹ-UTR. *MnHippo-b* possessed a 2067 bp ORF encoding a putative protein with 688 amino acids, a 136-bp 5ʹ-UTR, and a 5789-bp 3ʹ-UTR. *MnHippo-c* contained a 5ʹ-UTR of 136 bp, a 3ʹ-UTR of 5789 bp, and an ORF of 1971 bp encoding a polypeptide of 656 amino acids. The MWs of mature MnHippo-a, MnHippo-b, and MnHippo-c were estimated to be 79.77, 78.66 and 74.87 kDa, respectively, and their theoretical pIs were 5.35, 5.49, and 5.20, respectively.

**Figure 1:**
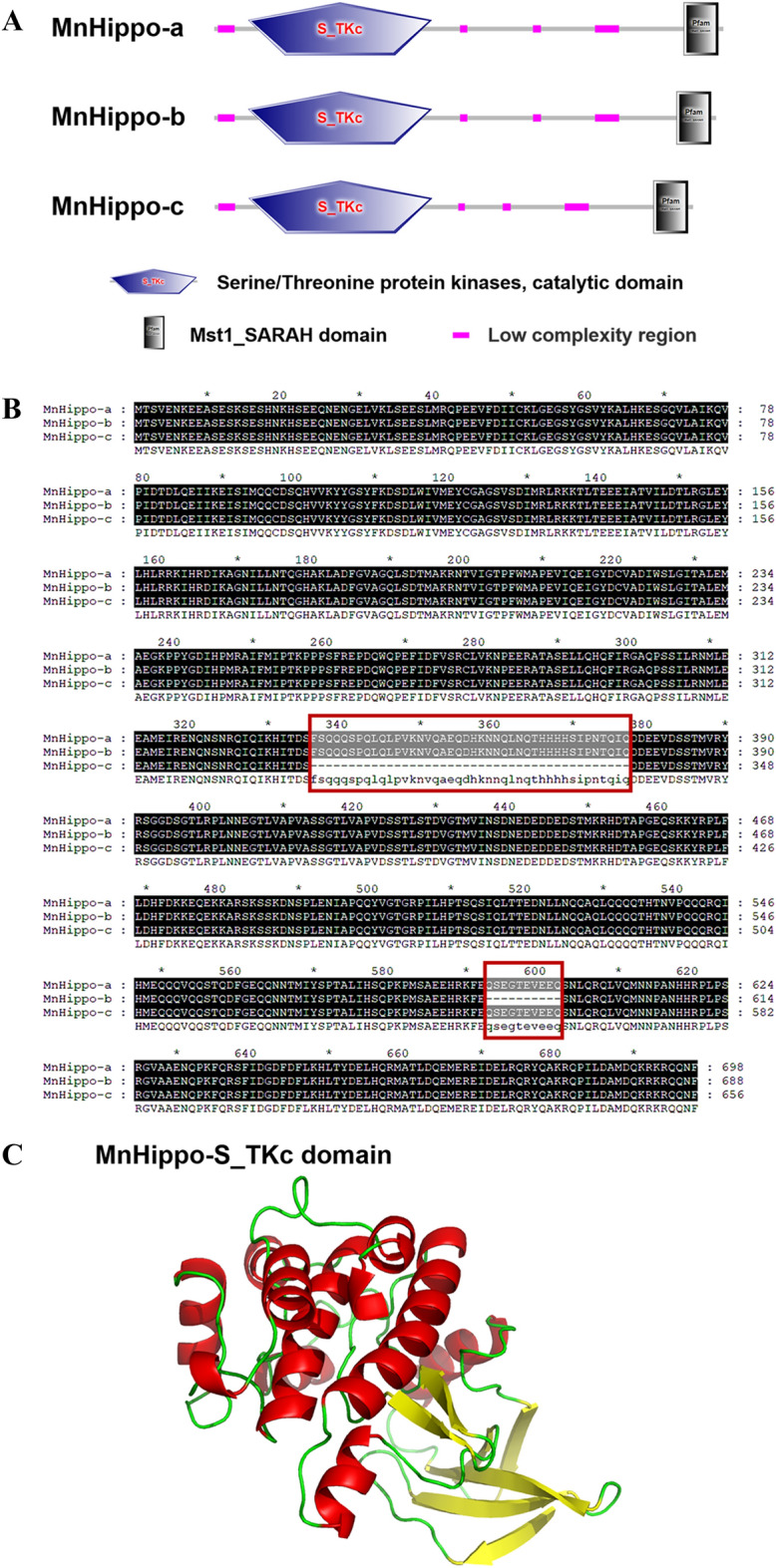
**Sequence analysis of MnHippo from *M. nipponense*.** **A** Domain topology of MnHippo-a, MnHippo-b, and MnHippo-c from *M. nipponense*. **B** Multiple alignment of amino acid sequences of the three MnHippo isoforms. The black shaded regions represent the identical residues. Other conserved but not consensus amino acids are shaded in grey. **C** The three-dimensional structure of the S_TKc domain in MnHippo was predicted using the SWISS-MODEL program and PyMOL.

The three MnHippo isoforms possessed the characteristic motifs of the serine/threonine protein kinase (STPK) family, including a serine/threonine protein kinase catalytic domain (S_TKc, position 47–298, 252 amino acids) and an Mst1_SARAH (Sav/Rassf/Hpo) domain (48 amino acids) (Figure 1A). When ClustalX was used to align the amino acid sequences of the three transcript isoforms, MnHippo-b and MnHippo-c had partially deletions compared with the complete amino acid sequence of MnHippo-a. MnHippo-b lacked the sequence QSEGTEVEEQ (10 amino acids), whereas MnHippo-c lacked the sequence FSQQQSPQLQLPVKNVQAEQDHKNNQLNQTHHHHSIPNTQIQ (42 amino acids) (Figure 1B). The function of a protein is defined by its structure. Therefore, STPK3 (SMTL ID: 4lgd.4) from *Homo sapiens* [27] was used as a template structure for homology modelling of the tertiary structure of S_TKc of MnHippo (Figure 1C). The S_TKc of MnHippo was composed of 11 α-helices and seven β-sheets and was structurally similar to STPK3 and human Mst2 kinase. This finding suggested the possible functional resemblance between MnHippo and other STPKs.

### Amplification of the *MnHippo* genomic sequence

**Figure 2:**
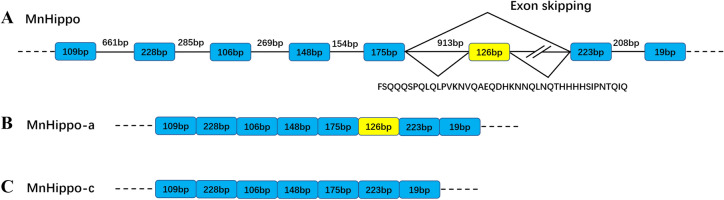
**Partial genomic structure of *MnHippo* from *M. nipponense*.** The exons are shown in blue boxes, the introns are indicated by horizontal lines, and unknown intron sequences are marked with double slashes. Intron–exon boundaries were determined by sequencing. *MnHippo-a* and *MnHippo-c* were derived from alternative splicing. *MnHippo-c* was generated by exon skipping.

PCR was used to amplify the partial genomic DNA sequence of *MnHippo* to investigate how the three isoforms of *MnHippo* were formed. The exon–intron boundaries of the genomic sequence of *MnHippo* were GT and AG at the 5ʹ and 3ʹ splice sites, respectively. The results of genome amplification suggested that *MnHippo-a* and *MnHippo-c* were derived from alternative splicing. *MnHippo-c* was generated by exon skipping (Figure 2).

### Sequence homology and phylogenetic analysis

**Figure 3:**
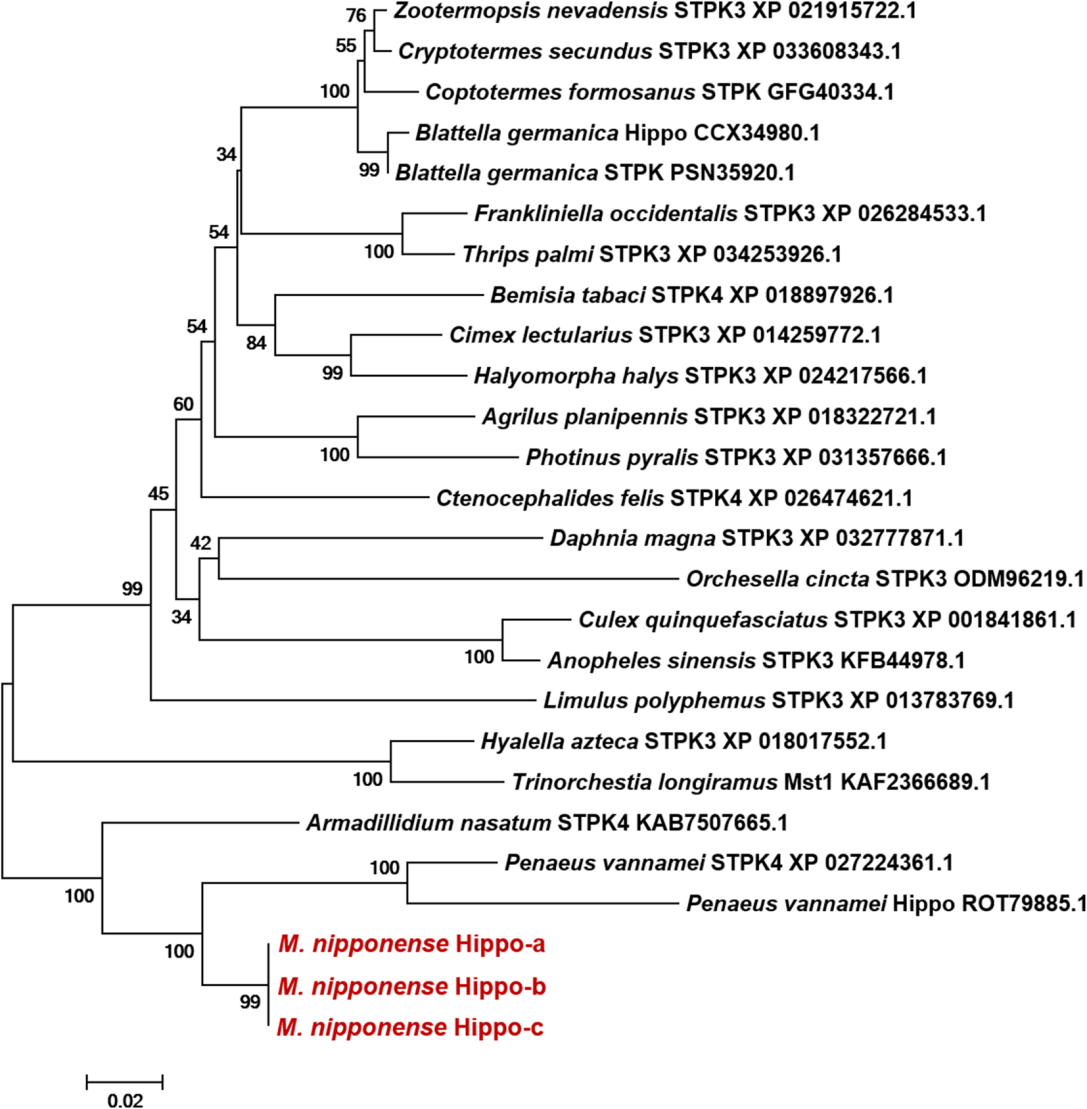
**Phylogenetic tree analysis of 26 STPK family members from different species.** A phylogenetic tree was constructed by the NJ algorithm in MEGA 7.0. The numbers at the nodes represent the bootstrap values. Three MnHippo isoforms are marked by red. *Agrilus planipennis* STPK3 (XP_018322721.1); *Anopheles sinensis* STPK3 (KFB44978.1); *A. nasatum* STPK4 (KAB7507665.1); *Bemisia tabaci* STPK4 (XP_018897926.1); *Blattella germanica* Hippo (CCX34980.1); *B. germanica* STPK (PSN35920.1); *Cimex lectularius* STPK3 (XP_014259772.1); *Coptotermes formosanus* STPK (GFG40334.1); *Cryptotermes secundus* STPK3 (XP_033608343.1); *Ctenocephalides felis* STPK4 (XP_026474621.1); *Culex quinquefasciatus* STPK3 (XP_001841861.1); *Daphnia magna* STPK3 (XP_032777871.1); *Frankliniella occidentalis* STPK3 (XP_026284533.1); *Halyomorpha halys* STPK3 (XP_024217566.1); *Hyalella azteca* STPK3 (XP_018017552.1); *Limulus polyphemus* STPK3 (XP_013783769.1); *Orchesella cincta* STPK3 (ODM96219.1); *P. vannamei* STPK4 (XP_027224361.1); *P. vannamei* Hippo (ROT79885.1); *Photinus pyralis* STPK3 (XP_031357666.1); *Thrips palmi* STPK3 (XP_034253926.1); *Trinorchestia longiramus* Mst1 (KAF2366689.1); *Zootermopsis nevadensis* STPK3 (XP_021915722.1).

Multiple protein sequence alignment revealed relatively highly conserved sequences between MnHippo-a and some STPKs in other species. The results showed a similarity of approximately 83.8% with STPK4 of *Penaeus vannamei*, 75.8% with Hippo of *P. vannamei*, 63.9% with STPK4 of *Armadillidium nasatum*, and 61.3% with STPK3 of *Hyalella azteca*. A phylogenetic tree was constructed based on the deduced amino acid sequence by using MEGA7 (Figure 3). Phylogenetic analysis showed that the three MnHippo isoforms (MnHippo-a, MnHippo-b and MnHippo-c) from *M. nipponense* shared the closest relationship with STPK4 and Hippo of *P. vannamei*. The amino acid sequence showed some highly conserved sites.

### Tissue distribution of *MnHippo*

**Figure 4:**
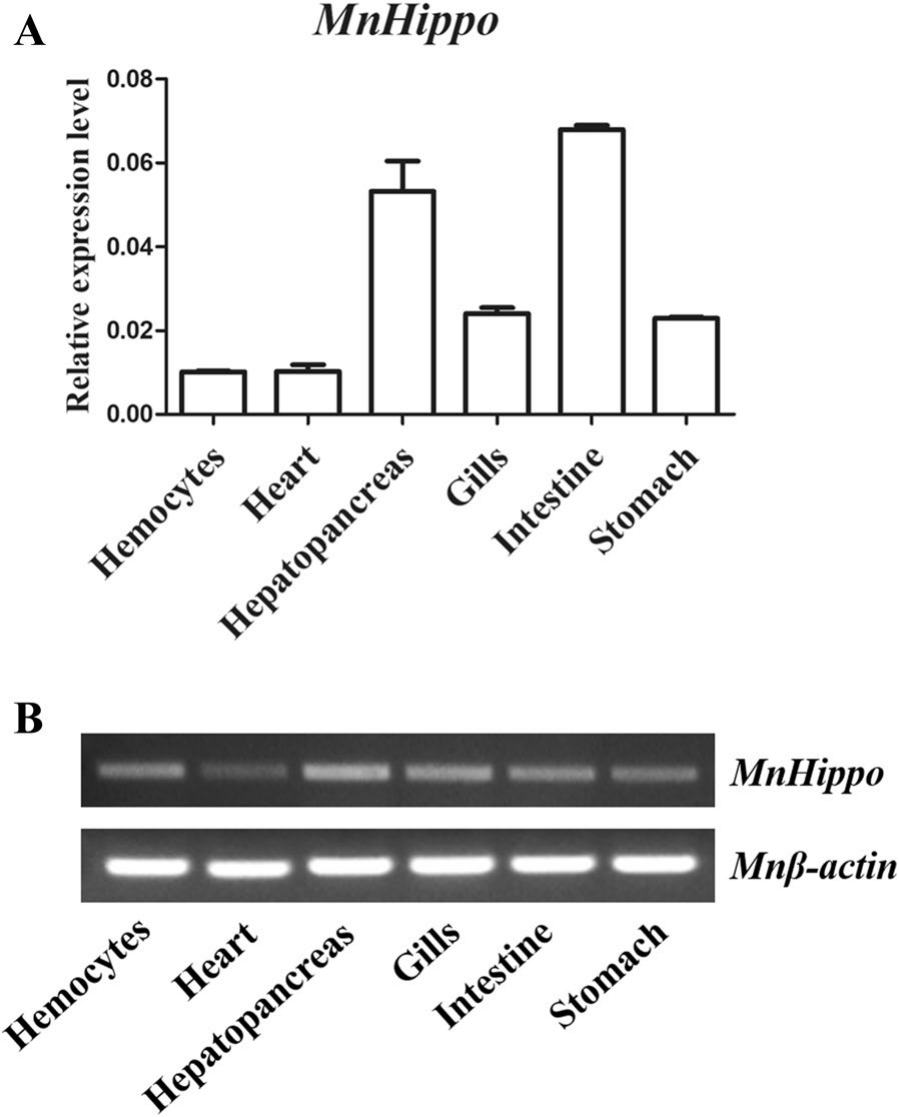
**Tissue distribution of *MnHippo*.** The mRNA expression level of *MnHippo* in different tissues (haemocytes, heart, hepatopancreas, gills, intestine, and stomach) was detected by qRT-PCR (**A**) and SqRT-PCR (**B**). *β*-Actin was used as a reference gene. The values were shown as the mean ± S.D. (*N* = 5).

The sequence amplified by the specific primers used for qRT-PCR was in the common region of the three isoforms, so MnHippo was used to represent the sum of three isoforms (MnHippo-a, MnHippo-b and MnHippo-c). The mRNA transcripts of *MnHippo* were detected in a wide range of tissues in uninfected *M. nipponense*, including haemocytes, heart, hepatopancreas, gill, intestine, and stomach. *MnHippo* expression was mostly detected in the intestine and hepatopancreas, and lower expression of this gene was detected in the heart and haemocytes (Figure 4).

### Temporal change in *MnHippo* mRNA after bacterial challenge

**Figure 5:**
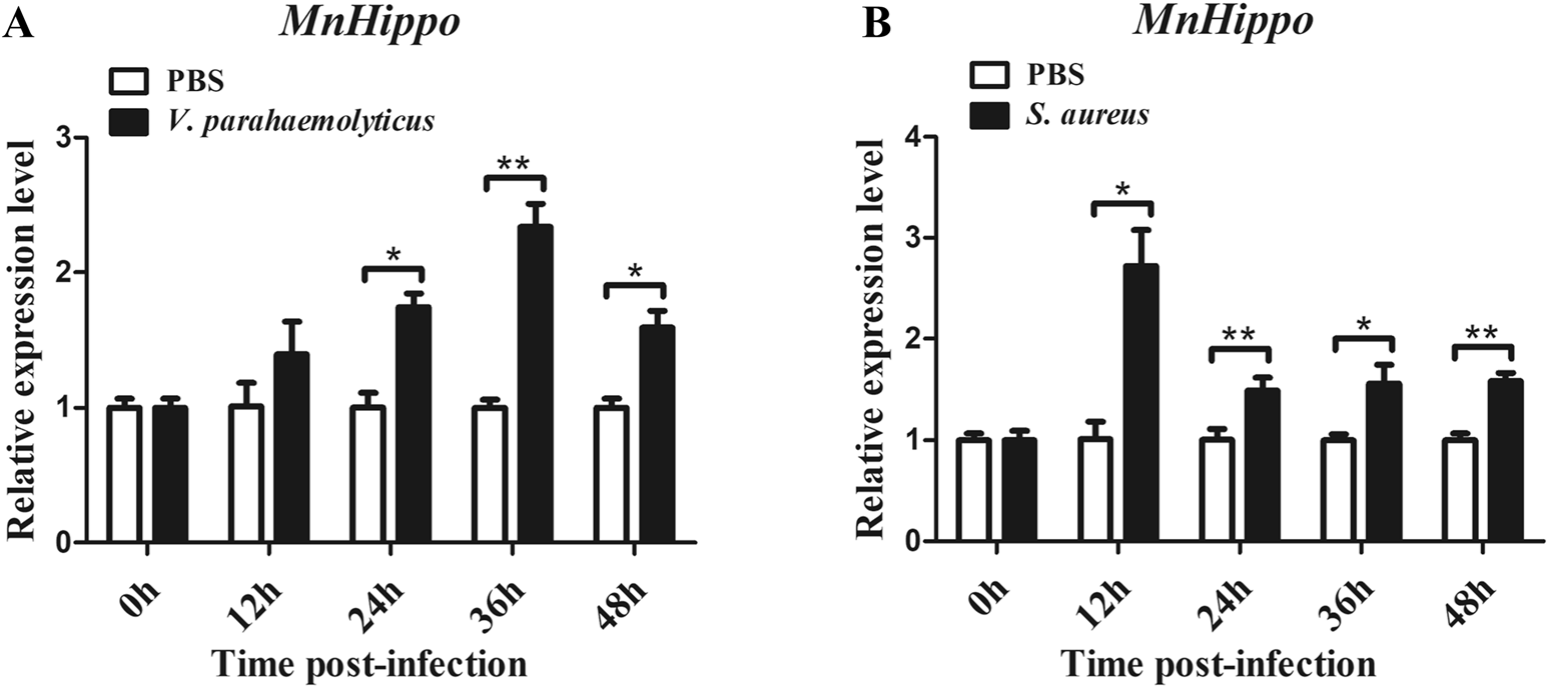
**Temporal mRNA expression of *MnHippo* in haemocytes at 0, 12, 24, 36, and 48 h after *V. parahaemolyticus* (A) and *S. aureus* (B) challenge.** Samples injected with PBS were adopted as controls, and *β*-actin was used as an internal reference gene. Vertical bars represent the mean ± S.D. (*N* = 5) (* *P* < 0.05, ** *P* < 0.01).

The temporal mRNA expression profile of *MnHippo* in the haemocytes of *M. nipponense* following *V. parahaemolyticus* and *S. aureus* challenge is presented in Figure 5. *MnHippo* expression was induced and gradually upregulated during the first 12 h after *V. parahaemolyticus* challenge. The expression peaked at 36 h and then decreased at 48 h (Figure 5A). After *S. aureus* infection, the level of *MnHippo* transcripts increased rapidly at 12 h and decreased slightly from 24 to 48 h, but it was still higher than the original expression level (Figure 5B). In the control group, the mRNA expression of *MnHippo* in haemocytes did not change significantly from 0 to 48 h (after PBS injection).

### *MnHippo* regulates the expression of antimicrobial peptides (AMPs) in response to stimulation

**Figure 6:**
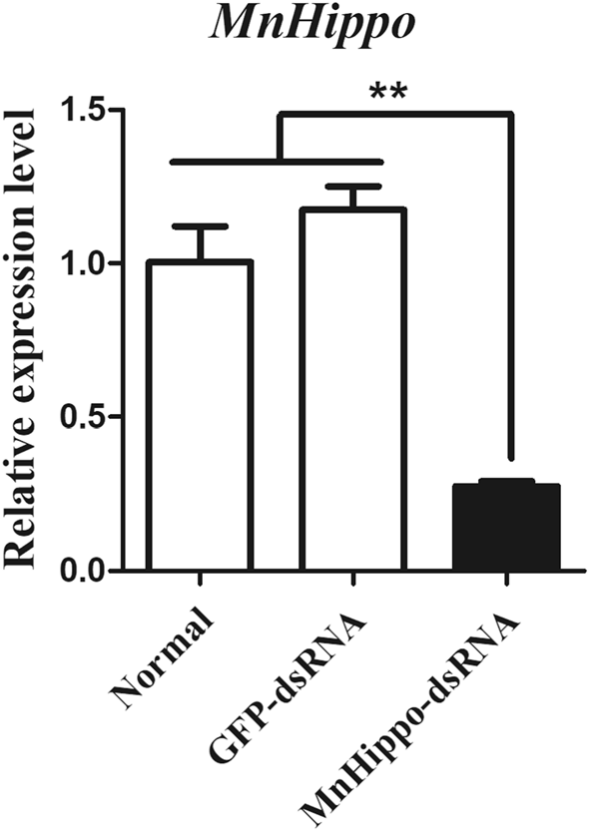
**Efficiency of *MnHippo* knockdown by dsRNA interference.** Thirty micrograms of *MnHippo*-dsRNA was injected into each prawn. The normal group and GFP-dsRNA injection group served as control groups. Haemocytes of five prawns from each group were sampled at 36 h post-injection to detect the RNAi efficiency by qRT-PCR. *β*-Actin was used as the reference gene. Vertical bars represent the mean ± S.D. (*N* = 5). The asterisk indicates a significant difference between the *MnHippo*-dsRNA group and the GFP-dsRNA group (**P < 0.01).

**Figure 7:**
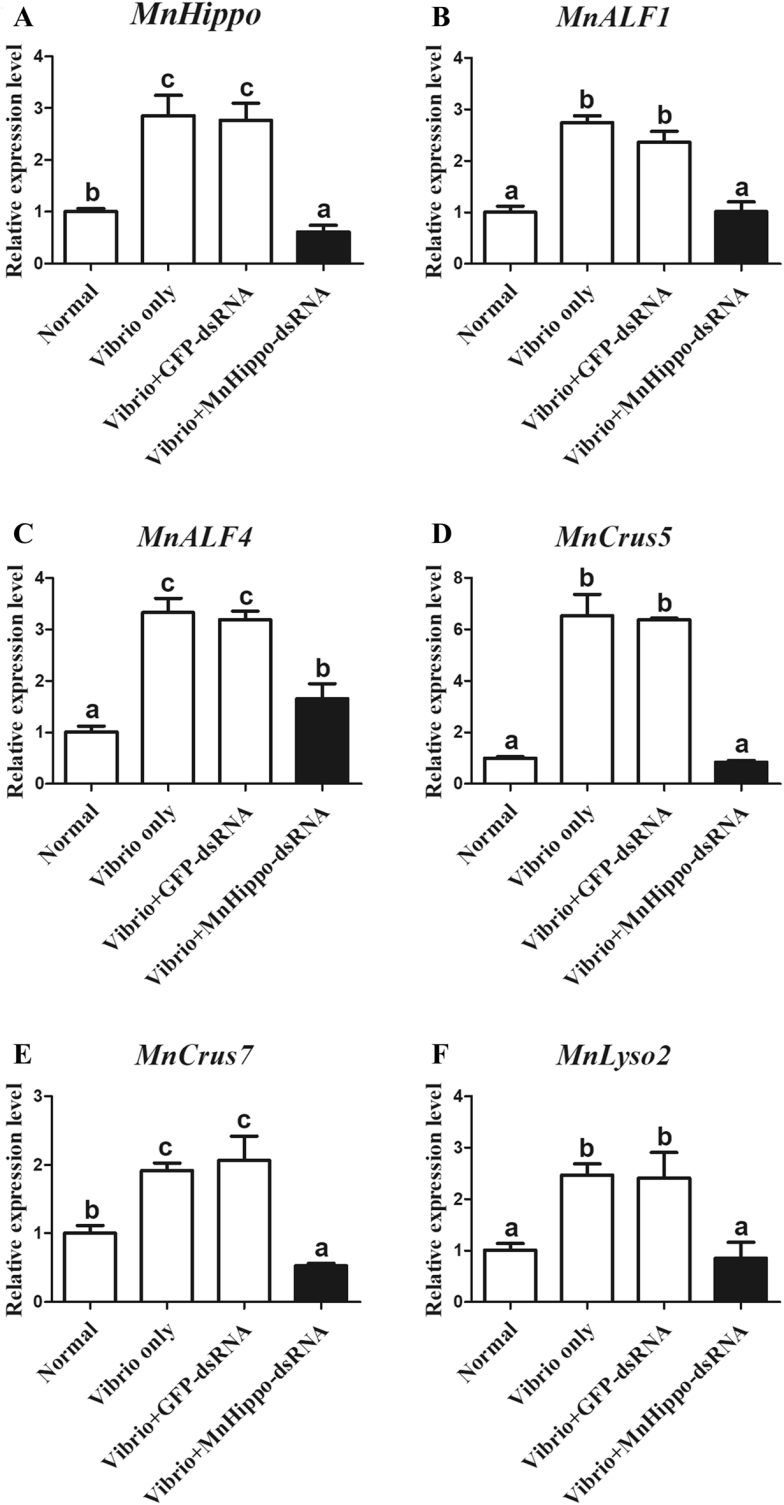
**Regulatory effect of *MnHippo* on the expression of *AMPs*.** **A** Sequence-specific *MnHippo*-dsRNA was injected into *V. parahaemolyticus*-infected prawns to inhibit *MnHippo* expression. **B**–**F** The mRNA expression levels of *MnALF1*, *MnALF4*, *MnCrus5*, *MnCrus7*, and *MnLyso2* after *V. parahaemolyticus* stimulation in the haemocytes of *MnHippo*-interfered prawns. Significant differences between the normal group and treated groups were subjected to one-way ANOVA. The different letters indicate significant differences compared with the other groups (*P* < 0.05).

**Figure 8:**
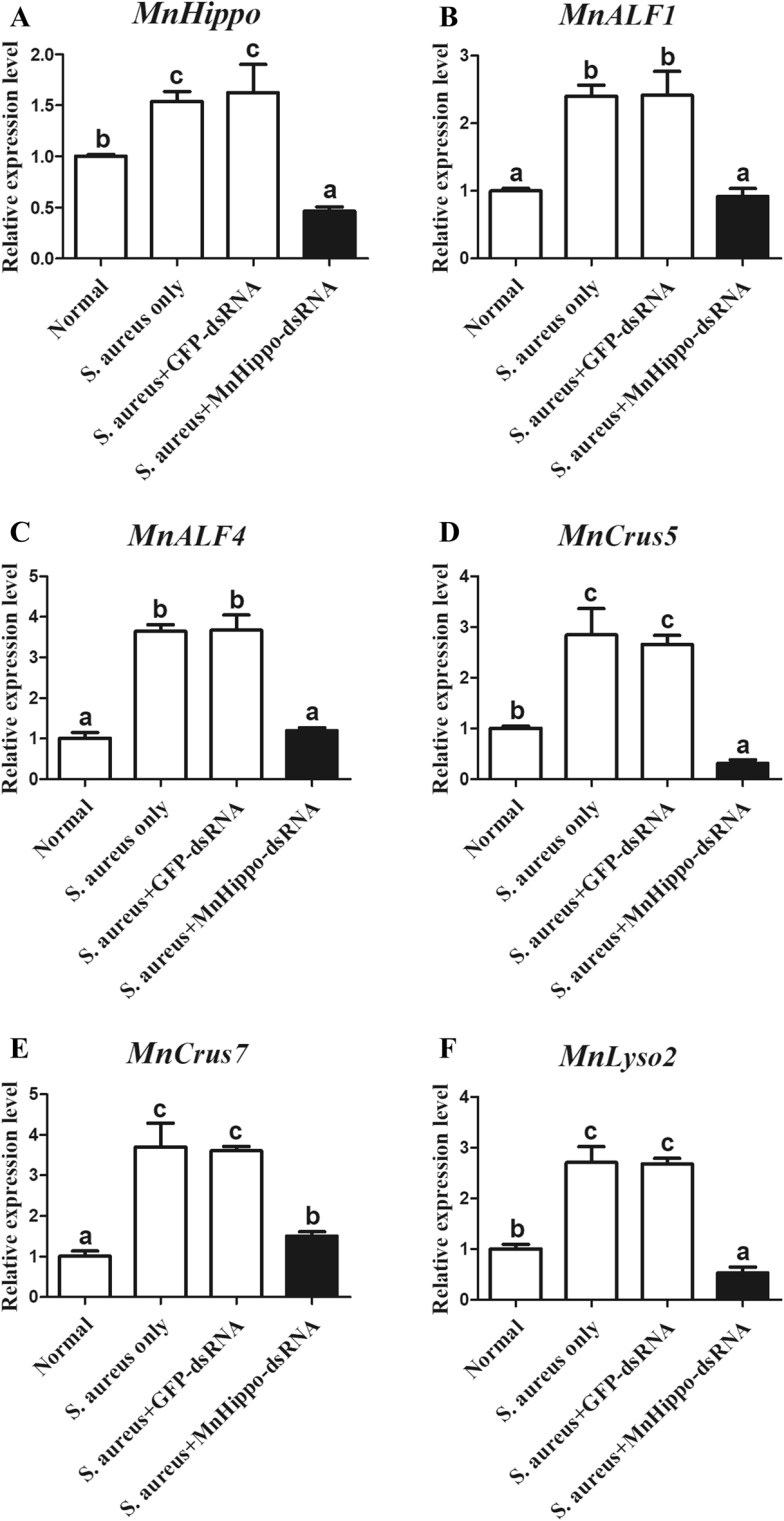
**Regulatory effect of *MnHippo* on the expression of *AMPs*.** **A** Sequence-specific *MnHippo*-dsRNA was injected into *S. aureus*-infected prawns to inhibit *MnHippo* expression. **B**–**F** The mRNA expression levels of *MnALF1*, *MnALF4*, *MnCrus5*, *MnCrus7*, and *MnLyso2* after *S. aureus* stimulation in the haemocytes of *MnHippo*-interfered prawns. The significant differences between the normal group and treated groups were subjected to one-way ANOVA. The different letters indicate significant differences compared with the other groups (*P* < 0.05).

In the RNAi experiments, *MnHippo*-dsRNA was used to silence *MnHippo* expression in the haemocytes of *M. nipponense* by approximately 72% (Figure 6). Following RNAi treatment and exposure to *V. parahaemolyticus* (Figure 7) and *S. aureus* (Figure 8) for 24 h, the expression levels of five AMPs, namely, *MnALF1*, *MnALF4*, *MnCrus5*, *MnCrus7*, and *MnLyso,* were detected using qRT-PCR. The results revealed that the transcription levels of the five AMPs were significantly downregulated (*P* < 0.05) after the effective knockdown of *MnHippo*. *MnCrus5* was strongly downregulated after *V. parahaemolyticus* or *S. aureus* stimulation. The expression levels of AMP genes in prawns treated with GFP-dsRNA showed no obvious differences compared with those in the control. These results suggested that AMPs might play a role in *MnHippo*-mediated defence against bacterial infection in *M. nipponense* haemocytes.

### *MnHippo* knockdown inhibits the clearance of bacteria in prawns

**Figure 9:**
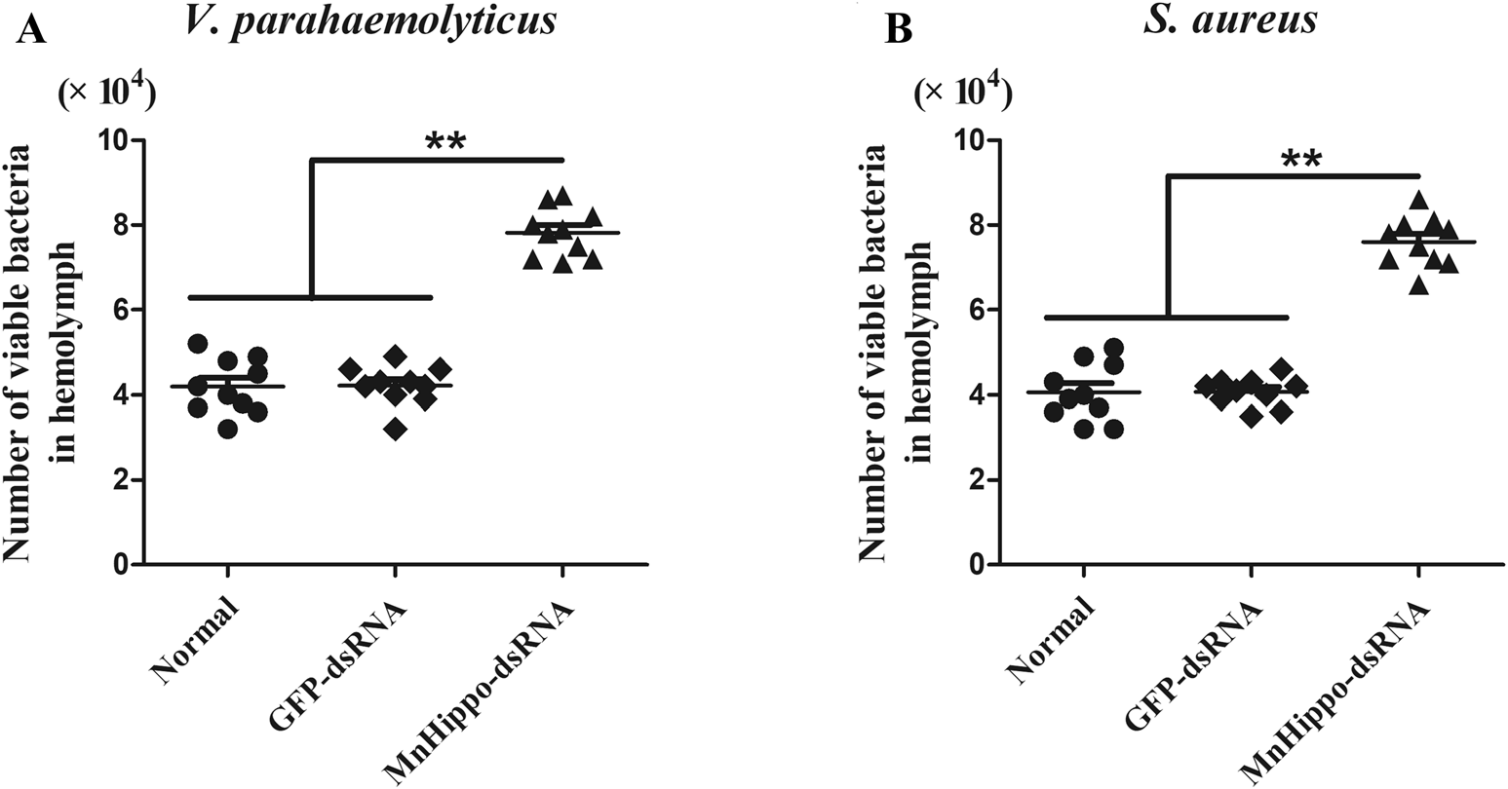
**Bacteria clearance assay in vivo.** *V. parahaemolyticus* (**A**) or *S. aureus* (**B**) was injected into normal or *MnHippo*-dsRNA/GFP-dsRNA-injected prawns. The same volume of haemolymph (500 μL) was extracted from prawns at 20 min after injection in different groups, diluted with sterile PBS, and cultured on LB medium plates at 37 °C overnight. The number of bacterial colonies was counted. The data are shown as the mean values ± S.D. (*N* = 10). The asterisks indicate significant differences (** *P* < 0.01).

The prawns were co-injected with *MnHippo*-dsRNA and bacteria (*V. parahaemolyticus* or *S. aureus*) to analyse whether *MnHippo* knockdown can affect the elimination of bacteria. As shown in Figs. 9A, B, the number of tested bacteria (*V. parahaemolyticus* or *S. aureus*) was significantly higher in the *MnHippo*-dsRNA group than in the control groups (normal and GFP-dsRNA injected groups) at 15 min post-injection. This finding indicated that silencing *MnHippo* inhibited bacterial clearance in prawns.

### Effects of *MnHippo* knockdown on the survival of challenged prawns

**Figure 10:**
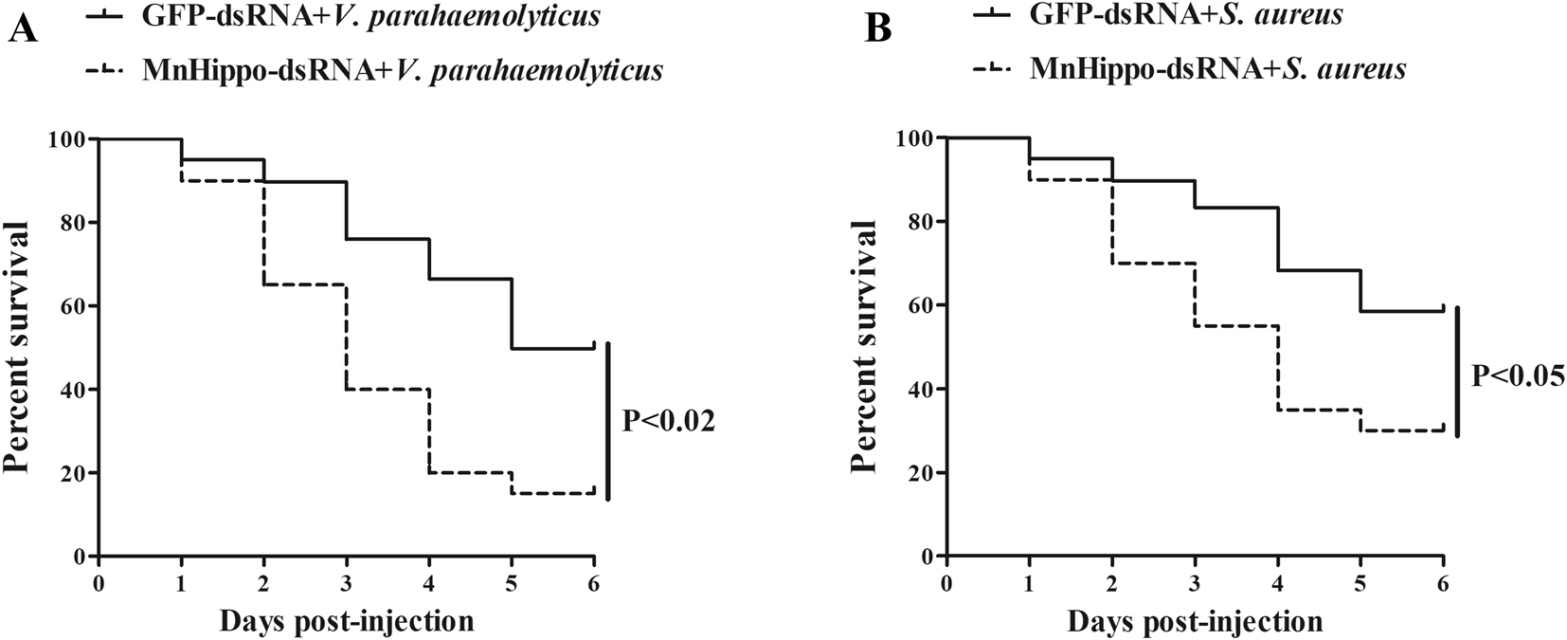
**Evaluation of prawn survival rate.** The prawns were co-injected with *MnHippo*-dsRNA or GFP-dsRNA and *V. parahaemolyticus* (**A**) or *S. aureus* (**B**). At various times (1–6 days) after infection, the survival rate of the prawns was examined.

Considering that *MnHippo* knockdown could obviously reduce the bacterial clearance rate, we further tested the survival rate after *MnHippo*-silenced prawns were infected with *V. parahaemolyticus* or *S. aureus*. The survival rate of prawns from 2 to 6 days after *MnHippo*-dsRNA plus *V. parahaemolyticus* or *S. aureus* injection was obviously lower than that in the control group (GFP-dsRNA plus *V. parahaemolyticus* or *S. aureus*) (Figure 10). The PBS group was used as a normal reference, and almost no prawns died in this group (data not shown). These data indicated that silencing *MnHippo* significantly reduced the survival rate of prawns infected with *V. parahaemolyticus* or *S. aureus*, thereby promoting their cumulative mortality.

## Discussion

Various studies have focused on the function and regulation of the Hippo signalling pathway in *Drosophila* and mammals. Many crucial components and new regulators of the Hippo pathway have been cloned and characterized. However, the current understanding of the Hippo pathway in crustaceans is still very limited, especially that of its functions related to defence against pathogen infection.

The Hippo pathway, which was named after *Hippo*, a *Drosophila* kinase-encoding gene, was independently shown to restrict tissue growth by several research groups more than ten years ago [5, 28]. In this study, a Hippo homologue with three isoforms (*MnHippo-a*, *MnHippo-b*, and *MnHippo-c*) was identified for the first time from the haemocytes of *M. nipponense*. The sequences of the three isoforms were highly similar. Compared with the amino acid sequence of MnHippo-a, the sequences of MnHippo-b and MnHippo-c showed partially deletions, with 10 and 42 amino acids deleted, respectively. *MnHippo-a* and *MnHippo-c* were derived from alternative splicing. Although the complete genome sequence of *MnHippo* was not yet available, we speculated that *MnHippo-b* was also formed by alternative splicing. However, the splicing method used to form *MnHippo-b* remains to be further studied.

Multiple sequence alignments revealed that the two missing parts in MnHippo-b and MnHippo-c were located in unknown regions, and the predicted protein domains of the three isoforms were identical. No signal peptide was found at the N-terminus of the MnHippo protein. The Hippo protein was speculated to be an intracellular protein. The mature MnHippo protein contained an S_TKc domain at the N-terminus and an Mst1_SARAH domain at the C-terminus, consistent with the other Hippo proteins of the Pacific white shrimp *P. vannamei* (ROT79885.1) and insects, such as *D. melanogaster* (NP_611427.1) and *A. nasatum* (KAB7507665.1). Further sequence analysis indicated that MnHippo could be a phosphotransferase and a member of the STPK subfamily. Protein kinases play roles in a variety of cellular processes, such as division, proliferation, apoptosis, and differentiation [29]. Protein phosphorylation is a reversible process mediated by protein kinase and phosphoprotein phosphatase and plays a crucial role in most cellular activities [30]. Phosphorylation usually results in functional changes in the target protein by altering enzyme activity, cell location, or interaction with other proteins [31]. In the Sav and Hpo families, the SARAH domain mediates signal transduction from Hpo via the Sav scaffolding protein to the downstream component Wts [32]. The Mst1_SARAH domain associates with Ras-association domain family 1 (Rassf1) and Rassf5 by forming a heterodimer, which mediates the apoptosis process [33]. MnHippo contains two characteristic domains that can phosphorylate downstream proteins to ensure normal signal transmission. Hippo mutants resulted in uncontrolled growth in various tissues owing to excessive cell proliferation and reduced apoptosis [34].

In uninfected adult *M. nipponense*, *MnHippo* was constitutively expressed in various tissues and highly expressed in the intestine and hepatopancreas. The intestine is a crucial organ for digestion, absorption, and nutrient exchange as well as for immunity [35]. In crustaceans, the intestinal barrier serves as the first line of host defence against environmental stress [36]. The hepatopancreas is an integrated organ of immunity and metabolism and is involved in the synthesis of immune and growth factors as well as in the maintenance of homeostasis in crustaceans [37, 38]. Therefore, the abundance of *MnHippo* in the two immune-related tissues indicated its potential immune defence function in *M. nipponense*. Although the expression of the *MnHippo* gene in the haemocytes of healthy prawns was lower than that in other tissues, haemocytes are one of the most critical cells involved in the innate immunity of crustaceans. In this regard, we selected haemocytes for immune stimulation experiments. In *Drosophila*, Gram-positive bacteria rather than Gram-negative bacteria induced the activation of the Hippo pathway [17]. Here, Gram-negative *V. parahaemolyticus* and Gram-positive *S. aureus* were selected to test whether they can activate the Hippo pathway in *M. nipponense*. After stimulation of *V. parahaemolyticus* or *S. aureus* via the ventral sinus, the expression of *MnHippo* was significantly upregulated. The expression level of *MnMOB1*, a central signal adaptor in the Hippo pathway, was also upregulated in the hepatopancreas, gills, and intestine of *M. nipponense* [39]. In the Chinese mitten crab *Eriocheir sinensis*, haemocytes infected with *S. aureus* or *V. parahaemolyticus* obviously increased the phosphorylation of *Es*Hpo at 0.5 and 1 h post-stimulation [40]. This result suggested that *MnHippo* might be involved in defence mechanisms against *V. parahaemolyticus* and *S. aureus*.

Prawns and other invertebrates rely almost entirely on innate immune systems, namely, humoral and cellular immunity, to defend themselves from pathogen invasion [41, 42]. The Toll signalling pathway is an essential cascade that plays a key role in the production of AMPs and is the main mechanism by which invertebrates eliminate bacteria and fungi [43, 44]. In *Drosophila*, Hippo–Yorkie signalling proved to be acutely activated by only Gram-positive bacteria. Inhibition of the Hippo pathway promoted Yorkie translocation and then regulated the expression of Cactus; the high expression of Cactus prevented the migration of Dorsal/Dif from the cytoplasm into the nucleus, thereby reducing the expression of AMPs [17]. This interesting study demonstrated the important contribution of the Hippo pathway and its crosstalk with the Toll pathway to innate immunity. We suspected that such an immune-related function for the Hippo pathway may also exist in other invertebrate species. In this regard, silencing of *MnHippo* was performed by specific dsRNA injection to characterize its role in the innate immunity of prawns. *MnHippo*-silenced haemocytes showed significantly low expression of several AMPs, such as *MnALF1*, *MnALF4*, *MnCrus5*, *MnCrus7*, and *MnLyso2*, which are Toll pathway target genes in crustaceans [44]. These results were consistent with a study on *MnMOB1*; in this work, the expression levels of *MnCrus* and *MnALF* were significantly downregulated when *MnMOB1* expression was knocked down after *S. aureus* and *V. parahaemolyticus* challenge [39]. In addition, *MnHippo* silencing weakened the clearance of *V. parahaemolyticus* and *S. aureus* in prawns. In the *V. parahaemolyticus-* or *S. aureus*-challenged group, the survival rate of the *MnHippo*-dsRNA group was significantly decreased from 2 to 6 days post-injection, corresponding to the *MnHippo* knockdown periods. However, the survival rates of non-related dsRNAs were 50% and 60% as a result of *V. parahaemolyticus* and *S. aureus* injection, respectively. Hence, *MnHippo* could resist bacterial invasion by regulating the transcription of some AMP genes. The detailed mechanism needs further in-depth research.

**Figure 11:**
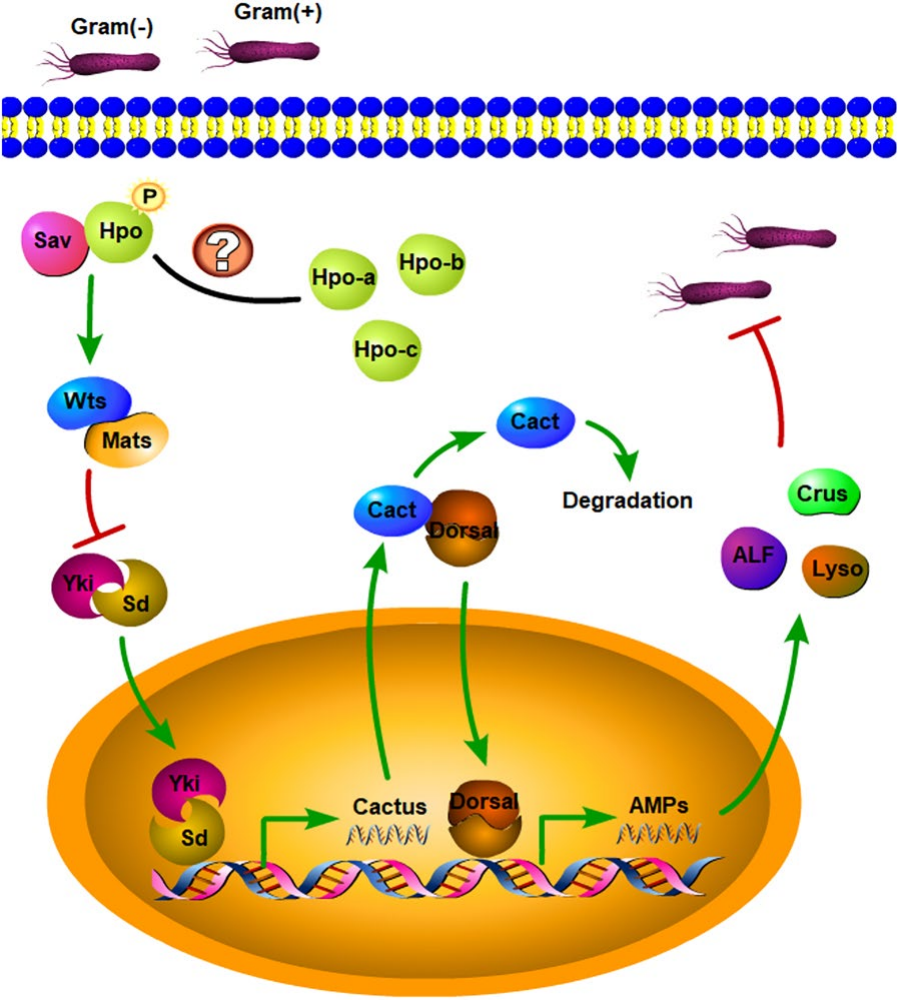
**Schematic representation of Hippo-regulated antimicrobial activities in oriental river prawns.** In *M. nipponense*, Hippo–Yorkie signalling was activated by Gram-positive and Gram-negative bacteria. The activation of this signalling axis excluded Yorkie from the nucleus. Inhibition of the Hippo pathway promoted Yorkie translocation, enabling Cactus transcription; the high expression of Cactus prevented the transfer of Dorsal/Dif from the cytoplasm into the nucleus, thereby reducing the expression of AMPs (such as ALF, crustin, and lysozyme).

In summary, a Hippo homologue with three isoforms was isolated and identified from *M. nipponense*. *MnHippo* expression in haemocytes was significantly induced by *V. parahaemolyticus* and *S. aureus* injection. The RNAi results validated the function of *MnHippo* in accelerating bacterial clearance and increasing the survival rate by regulating the expression of several AMPs. These results showed that *MnHippo* might be involved in the antibacterial immune defence of prawns. Further elucidating the involvement of the Hippo signalling pathway in innate immunity is of great significance (Figure 11).

## Supplementary Information

**Additional file 1. Nucleotide and deduced amino acid sequence of MnHippo-a from** ***M.. nipponense*.** The red letters indicate the start codon (ATG) and the stop codon (TAG). The S_TKc domain is underlined, and the italic letters indicate the Mst1_SARAH domain. Compared with MnHippo-a, MnHippo-b lacks the sequence marked in blue, and MnHippo-c lacks the sequence marked in green.
