# Supplementary figures and images for: A newly identified Hippo homologue from the oriental river prawn Macrobrachium nipponense is involved in the antimicrobial immune response
Source: Vet Res. 2021 Jun 2;52:76. doi: 10.1186/s13567-021-00945-7 (PMC8170997; doi:10.1186/s13567-021-00945-7)

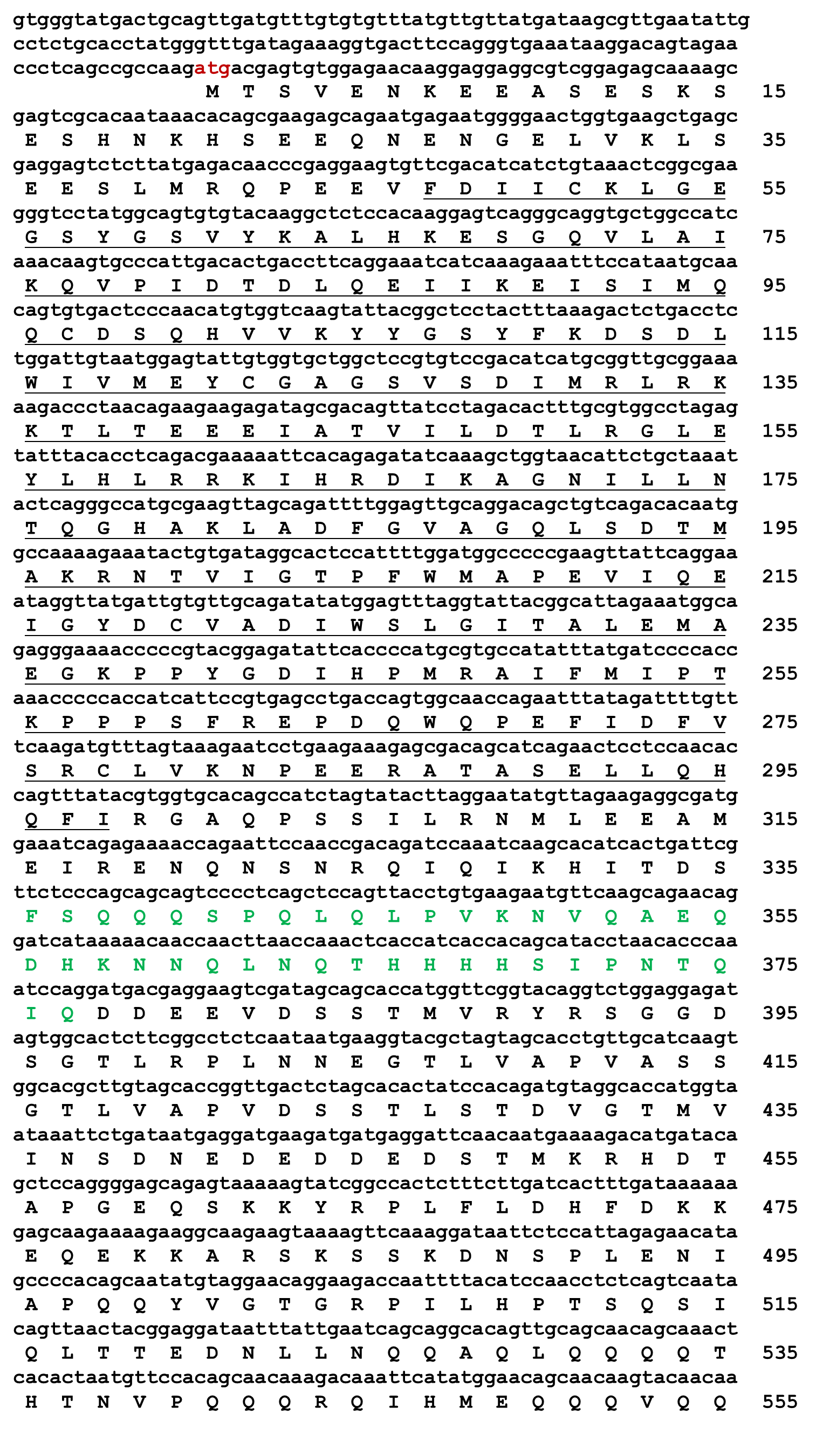


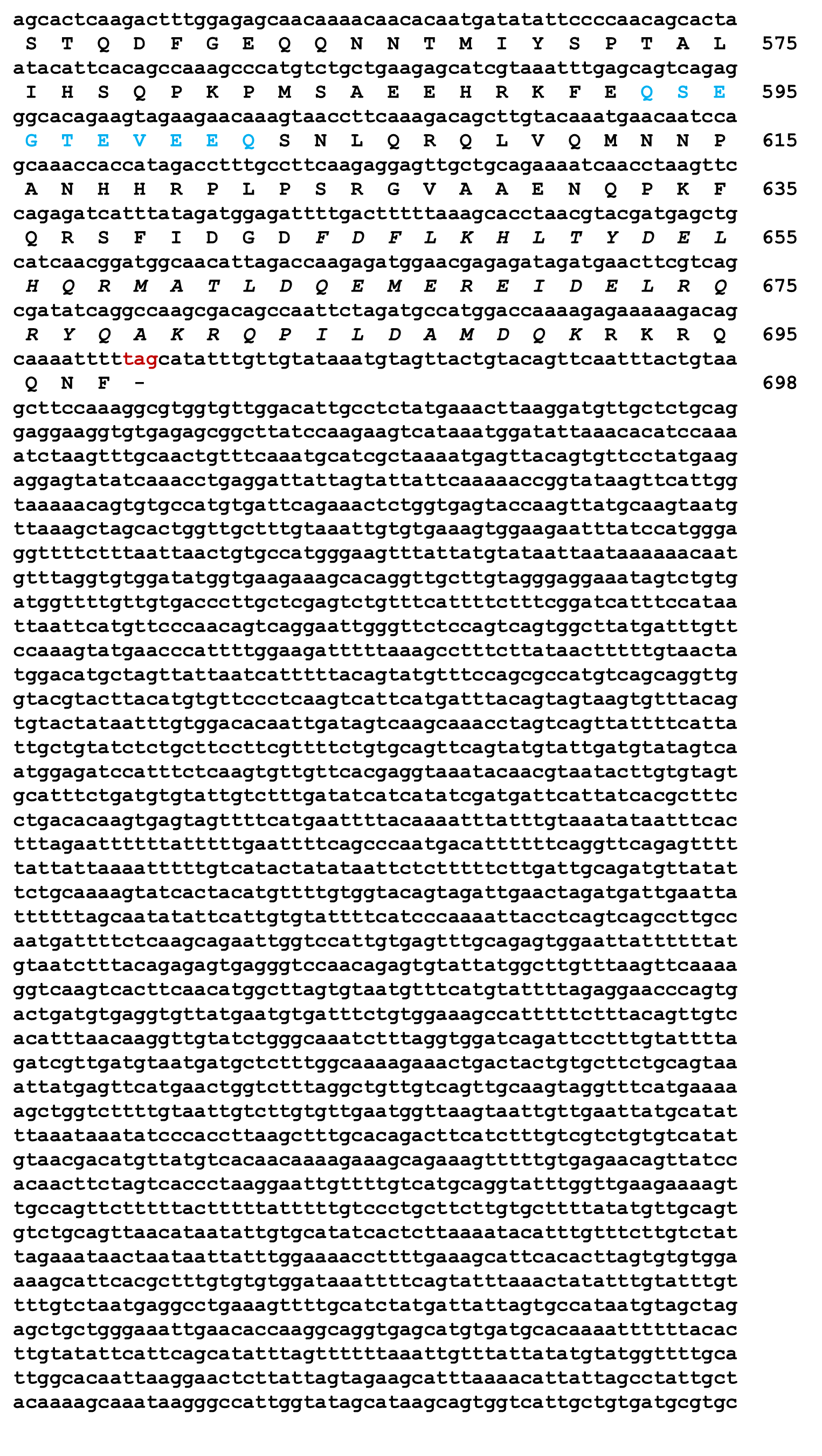


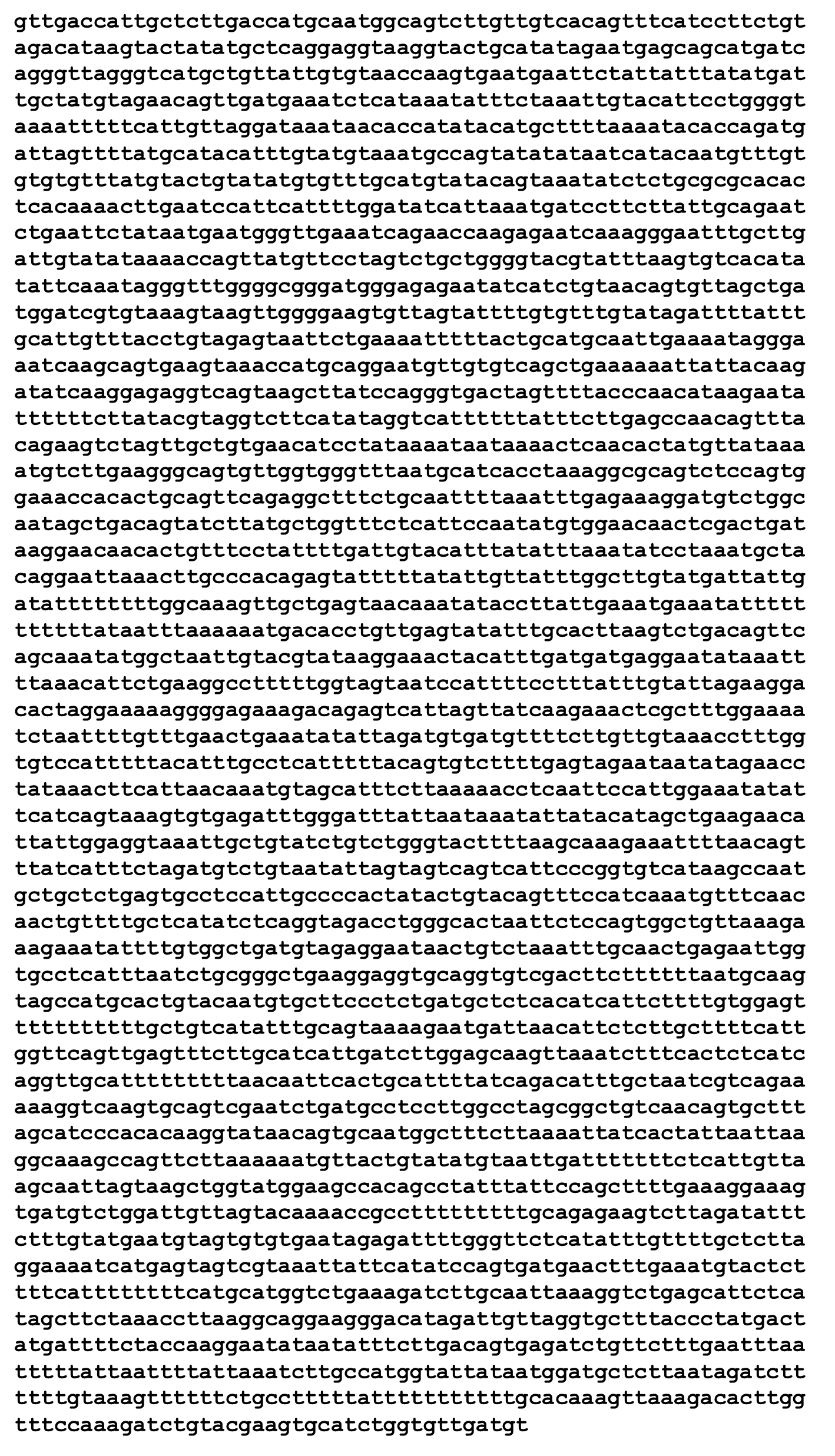

Supplement: Supplementary file 1 — Additional file 1. Nucleotide and deduced amino acid sequence of MnHippo-a from M.. nipponense. The red letters indicate the start codon (ATG) and the stop codon (TAG). The S_TKc domain is underlined, and the italic letters indicate the Mst1_SARAH domain. Compared with MnHippo-a, MnHippo-b lacks the sequence marked in blue, and MnHippo-c lacks the sequence marked in green. [file 13567_2021_945_MOESM1_ESM.docx]
